# Protective ventilation and negative fluid balance strategy in potential multi-organ donor patients

**DOI:** 10.1186/2197-425X-3-S1-A899

**Published:** 2015-10-01

**Authors:** M Sevilla Martínez, A Fernandez Carmona, MS Monsalve Alvarez de Cienfuegos, LC Navarro Guillamon, JM Perez Villares

**Affiliations:** Intensive Care Medicine, Virgen de las Nieves Hospital, Granada, Spain

## Objective

To describe our results of protective ventilation and negative fluid balance strategy in potential multi-organ donor patients.

## Material and Resources

A descriptive, retrospective study. We describe the results of a small series of potential donors (including older than 70 years) treated in single tertiary level center, from January to September 2013, in accordance with a strict protocol of protective ventilation, recruitment maneuvers, postural changes and negative fluid balance.

The collected variables were: Ventilatory parameters, central venous pressure, 24 hours fluid balance and information related to hemodynamic monitoring by transpulmonary thermodilution with Picco^®^ system (Pulse Contour Induced Cardiac Output).

## Results

13 potential donors were included. Mean age average was 46 years, 46,15% were male. Mean values of central venous pressure (CVP), extravascular lung water indexed (ELWI), a global diastolic volume indexed (GEDI) and stroke volume variation (SVV) at baseline were 7,08 cmH2O; 10,67 ml; 795,22 ml / m2 and 12,38% respectively. After application of the protocol, the mean values were: PVC 6,67 cmH2O; ELWI 8,13 ml; GEDI 683,38 ml / m2 and VVS 16,83%. Mean 24 hours fluids balance was -466,33 ml, mean ratio of partial pressure of arterial oxygen to the fraction of inspired oxygen (PaFiO2) improved of the initial value from 256,24 to 365,40 mmHg. Postural changes were made in 91,7% and alveolar recruitment in 83.3% of the patients, diuretics were required in 83.3% of them.

Of the 13 potential donors, 4 patients were lung donors. Within this subgroup, the final CVP decreased to 5,75 cmH2O; the ELWI to 7,33 ml and the GEDI to 660 ml / m2. 24 hours fluid balance was -1167,50 ml and PaFiO2 improved to 424,50 mmHg (initial < 300 mmhg).

Moreover of the 13 cases, there were also other donations: 3 hearts, 10 livers, 1 pancreas and 9 kidney donor.

## Conclusion

The depletion of fluids and adequate ventilation management is sure, improves lungs function and probably improves multi-organ donation results.

These measures allow rescue 4 lung donors which initially gasometric criteria contraindicates a possible donation.

